# Neglect of Psychological Care for Children with Cerebral Palsy and Their Families and Its Impact on Their Occupational Engagement in Saudi Arabia

**DOI:** 10.3390/medicina60081216

**Published:** 2024-07-27

**Authors:** Safaa M. Elkholi, Salwa S. Awad, Madawi H. Alotaibi

**Affiliations:** Department of Rehabilitation Sciences, College of Health and Rehabilitation Sciences, Princess Nourah bint Abdulrahman University, P.O. Box 84428, Riyadh 11671, Saudi Arabia

**Keywords:** cerebral palsy, parents, mental health care, occupational engagement, Saudi Arabia, support

## Abstract

*Background and Objectives*: Many children with (CP) and their families in Saudi Arabia struggle emotionally. Unfortunately, there have not been many studies conducted on how to help them with these challenges. This research aims to bring attention to this gap and to explore how a lack of proper mental health care might affect these children’s ability to participate in everyday activities. *Materials and Methods*: In this cross-sectional descriptive study, a survey was conducted between August and October 2023. A total of 300 parents of CP children from Saudi Arabia participated in the study. The impact of psychological care negligence on the occupational engagement of CP children and their families was assessed by designing a valid questionnaire. *Results*: A total of 300 parents of children with CP participated in this study. The majority of the sample, 71% of parents, said that their children did not receive any psychological care, and 59.7% of the participants said that their children did not even receive a referral to a psychologist. However, 60.3% of parents of children noticed a significant decline in the occupational performance of their children, and 65.7% predicted an improvement in their children’s performance with future psychological care. *Conclusions*: It is clear that there is a lack of awareness about the importance of mental health care for children with CP in Saudi Arabia. This lack of care hinders these children and their families’ occupational engagement and social participation.

## 1. Introduction

Cerebral palsy (CP) is a group of long-term developmental disorders affecting body movement, balance, and posture. It is a common cause of ill health and disability among children. CP can cause motor dysfunction and sensory, perceptual, cognitive, communication, learning, and behavioral impairments. Hereditary and acquired musculoskeletal impediments are frequently observed [1,2].

According to data from the World Health Organization (WHO), CP affects 10–40% of the population in low- and middle-income countries (LMICs). The prevalence of pre- and perinatal CP births is 3.4 per 1000 (95% CI 3.0–3.9) live births. When post-neonatal CP is taken into consideration, the birth prevalence for pre- and perinatal CP in high-income countries (HICs) is 1.5 per 1000 (95% CI 1.4–1.6) live births, and 1.6 per 1000 (95% CI 1.5–1.7) live births [3]. Recent worldwide population-based research has found that there are one to nearly four cases of CP for every 1000 live births [4]. A systematic review and meta-analysis conducted in Arabic-speaking countries confirmed that the incidence of CP was 1.8/1000 live births (95% CI: 1.2–2.5); when screened for the most prevalent motor type, spastic CP accounted for 59.8% (95% CI: 46.2–72.7) of the pooled estimates [5].

On the other hand, in Saudi Arabia, the prevalence of CP ranges from 1.2 to 2.5 per 1000 live births, and it has been estimated that in certain Saudi hospitals, CP accounts for 0.41% of live births and 2.3 cases per 1000 residents [6]. In developing countries, including Saudi Arabia, CP and other childhood disorders have not been given sufficient attention where there is limited evidence of pediatric disabilities, especially in the case of CP [7]. Despite this, a study conducted in 2011 by [8] found that the prevalence of CP in Saudi Arabia was 2.3/1000 inhabitants, and another study reported a 0.41% incidence of CP among 99,788 live births at the Riyadh Military Hospital [9]. In Saudi Arabia, there is evident variation in the reported incidence and prevalence of neurological disorders, primarily CP. According to recent research, 6.9 out of 1000 children have serious long-term neurological disorders. The most common neurological disorders and the most common chronic conditions in children are intellectual disability (ID) and CP, with rates of 2.7/1000 and 2.3/1000, respectively [8]. Children with disabilities are at a higher risk of mental health issues, which could develop into serious mental illnesses [10]. Negative social experiences can also contribute to emotional and behavioral disturbances [11]. Providing adequate support and care is crucial to preventing the development of more severe mental health issues. Tailored rehabilitation services can significantly improve a child’s skills and participation in activities [12,13]. This shows the magnitude of the problem and the urgent need to highlight it. Numerous studies and reports have been published globally on the lifetime cost of CP, which includes costs associated with productivity, social care, and healthcare [14]. Additional therapy is required for those with lower functioning abilities [15,16,17]. Families with children with CP encounter various social, developmental, and psychological hurdles. Taking care of a child with CP can affect parents’ health, finances, and overall quality of life [18]. The effects of the CP’s prevalence on society as a whole are profound in terms of social, economic, and public health issues. It can necessitate significant medical attention, therapy, assistive technology, and special education. According to [19], the disorder affects approximately 11.5 billion infants in the United States. A study investigated the global lifetime cost of CP, highlighting the costs associated with productivity due to limited employment opportunities, social care, and healthcare [14]. Specialized learning environments, therapies, and adaptive technologies may be necessary for children with CP. This may reduce access to mainstream education and put a burden on school resources [20]. Social interaction can be difficult for children with CP and their carers due to mobility and communication issues. This may reduce opportunities for social interaction and friendship building, as well as cause feelings of loneliness [21]. At the level of public health policies, CP requires early diagnosis and intervention programs that are crucial to maximize the potential and improve the quality of life for individuals with CP. Public infrastructure and transportation systems are required to be accessible for people with disabilities, including those with mobility limitations. Programs offering financial assistance, personal care services, and vocational training can empower individuals with CP to live independently and help them fully participate in their society [22]. It is also vital to invest in research to learn more about the causes of CP, develop better treatment options, and possibly even discover a cure. The impact of CP extends beyond the individual. Families of individuals with CP frequently bear heavy financial and psychological costs [23]. Furthermore, social opportunities for inclusion and participation may be further restricted by the lack of knowledge and comprehension.

Supporting caregivers is crucial to enable them to maintain their daily routines [24,25,26]. Regular evaluations of caregivers’ mental health are essential for the rehabilitation and integration of children with CP. Delivering psychological assistance is a vital component of caring for children who are living with this condition. For healthcare providers working with children with CP, it is crucial to address their unique needs and to keep a close eye on their development. Chronic impairments can significantly impact early childhood development, and school can be particularly challenging. Societal attitudes can sometimes discourage families from seeking psychological support, even though it can be incredibly beneficial for the children and their loved ones [27]. This creates barriers for children and caregivers to participate in social activities [28,29]. However, providing appropriate psychological care for children with CP can significantly improve their quality of life and that of their families. The literature focuses on two main factors that control how well parents of children with disabilities deal with the hindrances they face. These are protective and risk factors [30,31]. The protective factors that support resilience and help parents cope with stress are personal characteristics such as positiveness, self-efficacy, a positive outlook, and flexible thinking, which are all important. Effective coping mechanisms like problem solving, seeking support, and emotional regulation help parents to navigate difficult situations. A family network of family, friends, therapists, and disability advocacy groups can make a big difference. A secure financial condition allows parents to focus on their child’s needs [32]. In contrast, risk factors that can thwart parental resilience and increase their stress can include the severity of the child’s disability. The higher the child’s needs, the more stress parents may experience. Weaker social support can make it difficult for parents to cope. Parents may be more susceptible to anxiety or depression due to the stress of caring for a child with a disability. Difficulties in obtaining therapy, equipment, or financial assistance can add to the burden [33]. Understanding these factors can help parents to identify areas where they can build resilience. It can also inform support programs designed to assist families with children with disabilities [34]. This study explores the parental understanding of the significance of psychological health care for their children with CP and how it can affect their children’s occupational performance.

## 2. Materials and Methods

### 2.1. Study Design and Participants

A descriptive cross-sectional study was carried out between August and October 2023. The study utilized a quantitative method in which parents of CP children completed a self-administered questionnaire. The questionnaire was created to measure the impact of psychological care negligence on the occupational engagement of CP children and their families. Five experts reviewed the questionnaire to assess its validity, and pilot testing was carried out by giving the questionnaire to 30 parents to assess its reliability. Following pilot testing, the online questionnaire was sent through emails and social media to all rehabilitation centers and CP parents’ social groups in Saudi Arabia. The sample was selected using a convenient sampling method. The sample size for this study was estimated to be 341 parents of children with CP. The sample size was estimated based on a 5% margin of error and 95% confidence level for a total population size of 2968 children with CP in Saudi Arabia [34]. In this study, 300 parents of CP children voluntarily participated by answering an online questionnaire. Before responding to the questionnaire, participants were provided with explicit and detailed instructions and information on how to fill in the questionnaire, as well as further explanations on aspects such as confidentiality and that the research would be used only for research purposes. The participants in the study gave their consent to be informed about the study’s purpose.

### 2.2. Inclusion Criteria

Parents/caregivers of children with CP were invited to participate in the study based on the following inclusion criteria: CP children aged 5 to 15 years old, living in Saudi Arabia.

### 2.3. Exclusion Criteria

The following were the exclusion criteria: adults, children with muscular dystrophy disease, autistic children, children with Down syndrome.

### 2.4. Procedure

An online questionnaire was created in the Arabic language using a Google form. Five experts with varying degrees of experience in the field of rehabilitation and clinical psychology validated the questionnaire. After evaluating the questionnaire, they suggested some changes that were made by the authors. The face and content validities of the survey were tested by the experts. A pilot sample of thirty parents was then given the first draft of the questionnaire in order to evaluate its reliability. When the data were examined for internal consistency, a high level of internal consistency was indicated by a Cronbach’s alpha coefficient value of 0.77.

The first part of the questionnaire included personal and demographic data of the caregivers, such as their relationship to the child, gender, educational level, and employment status. Then, questions about the CP child were asked regarding their age, sex, and diagnosis. The second part of the questionnaire identified the caregiver’s awareness and understanding of psychiatric care and its potential social stigma. It then explored their experience with accessing psychiatric services for their child, including any past referrals to psychologists.

Following this, the questionnaire delved into the child’s cognitive abilities. Caregivers were asked if they had received a full explanation of their child’s intellectual disability and their perception of the link between CP and cognition. Additionally, they were asked to observe and report any perceived decline in their child’s cognitive abilities during social interaction.

The questionnaire then shifted focus to the impact of the child’s condition on daily activities. Caregivers were asked to assess any limitations their child faced and whether they believed these limitations stemmed primarily from cognitive challenges.

To investigate the perceived benefits of psychiatric care, the questionnaire inquired about any improvements observed in the child’s behavior following access to such services. Parents were also asked if they believed their child’s overall performance could benefit from psychiatric intervention, and whether they felt the need for psychological support to better understand their child’s behavior.

Finally, the questionnaire explored the impact of the child’s condition on the caregiver’s life. It asked about parental employment and any work attendance issues related to caring for their child. It further investigated their level of satisfaction with their work–life balance and the financial strain, if any, caused by their child’s condition. The questionnaire concluded by exploring how the child’s condition affected the caregiver’s social life, occupation, and interests.

### 2.5. Statistical Analysis

We used SPSS version 25 (Statistical Package for the Social Sciences) by IBM for Windows to analyze the data. We used a descriptive statistical analysis (frequency and percentage). We used the Pearson correlation coefficient to determine the degree of correlation between the resolution and phrases axes, which included all the words used by parents and the total score of the questionnaire. The reliability of the study tool was tested using the Cronbach alpha coefficient (Cronbach’s alpha).

### 2.6. Data Management

To protect the privacy of all participants, their data was coded. Only researchers involved in the study had access to the secure computer files where participants’ information was stored.

### 2.7. Ethical Considerations

This study was conducted with the approval of the Institutional Review Board Committee of PNU (23-0545). Participants were required to consent before completing the questionnaire and were informed that they could withdraw at any time. Before participating in the study, participants were informed about the purpose of the research and the confidentiality and anonymity of their identity. The survey method did not pose any physical or psychological risks to the participants.

## 3. Results

A total of 300 parents of children with CP in the age range of 5–15 years old participated in the study. Of these, 235 (78%) were women and 65 (21%) were men, with a majority holding a higher degree of education but unemployed. The data showed that nearly two-thirds of the primary caregivers (64%) were mothers. The data are shown in Table 1.

The results showed that 42% of the study participants were unaware of the meaning of psychological care. However, a significant portion of the sample was familiar with it.

### 3.1. Receiving Mental Health Care

Most respondents (71%) stated that their children had not received psychological care in the past, while 29% reported that their children had received such care. The study also found that 59.7% of the sample had never been referred to a psychologist. Additionally, 59% of the participants did not believe that seeking psychological care for their child would lead to social stigma, while 41% thought that it might.

### 3.2. Child’s Cognitive Ability

Additionally, more than half of the sample had not received a full explanation of their child’s mental abilities. Most parents (64%) believed that the CP disease affected their child’s intellectual capabilities. Nearly half of the sample (48.3%) reported a decrease in their child’s intellectual capabilities, which was evident during communication with others. However, 50.7% did not notice such a decrease. Most parents (60.3%) thought that their child faced difficulties while performing intellectual activities, and 55% believed that occupational performance was related to their child’s intellectual abilities.

Interestingly, 70% of the participants saw improvement in their children due to psychiatric care. Additionally, 65% of the sample believed that psychological care would improve their child’s functional abilities, while 43% did not. Furthermore, 50.3% of participants believed that they needed a psychologist’s help to communicate with their child better.

### 3.3. The Impact of a Child’s Condition on the Caregiver’s Life

Out of all the participants, a large portion were employees, accounting for 45.3%. Most of these employees (51%) stated that their children with CP impacted their ability to attend work. Regarding their occupational and domestic roles, 55% of the sample felt satisfied as a mother/father/worker, while 45% felt unfulfilled. About half of the sample (49.3%) believed that their child’s condition was a financial burden on the family. Based on the results, nearly half of the sample believed that the state of their child affected their social life, including social visits and practicing hobbies. The detailed results are presented in Table 2.

## 4. Discussion

The main objective of this study was to investigate the understanding of the significance of psychological health care for children with CP and its impact on children’s occupational performance. The study examined the degree of awareness of the importance of psychological care and how neglecting it affects children’s occupational engagement, productivity, social participation, and satisfaction. The study found that 64% of primary caregivers for children with CP in Saudi Arabia were mothers. This is consistent with the fact that mothers usually serve as the primary caregivers for their children. However, the weight of caring for a child with a disability can make it difficult for them to participate in adequate social activities. A previous study suggested that mothers who take better care of their children are less likely to be engaged in social participation [35].

Additionally, the study showed that 54.7% of children’s caregivers were unemployed, consistent with earlier studies [36,37,38]. This may be due to the lack of childcare facilities for children with disabilities in some nations, including Saudi Arabia [37]. Of the employed caregivers, 23.3% reported that their attendance at work had been impacted by their caregiving responsibilities.

Mothers were found to be more likely to cut back on their work hours or to stop working altogether as the severity of their child’s disability increased [39,40,41,42]. Despite the increasing attention given to gender equality, women still tend to be the primary caregivers for children. As a result, they may struggle to balance their work demands with their caregiving duties [43,44].

Research shows that mental health issues are common among children with CP [45,46,47]. One review study discovered that one in three children with CP experienced mental health problems [48]. Recent evaluations of children with CP at school entry and pre-adolescence have demonstrated an increase in the occurrence of psychiatric disorders, mainly emotional disorders [49,50], with rates increasing from 57% to 79%. Despite the growing need for psychological care for these children, the current study found that caregivers have limited knowledge of psychiatric/psychological care, with 65.7% of respondents indicating that mental health care will not improve their child’s performance. This could explain why these children have limited access to psychiatric care, as a significant number of participants (59.7%) reported that their children had never been referred to mental health services during their therapy. A recent study conducted by [51] in 2022 showed that 87% of participants preferred to consult with a physician rather than a mental health specialist for psychological concerns. Nutritionists, family members, herbalists, and social media were among the top five sources of information for treating psychological problems. A growing number of participants (41.0%) in the current study associate seeking psychological treatment for their child with a social stigma, which could explain their reluctance to seek mental health care for their children with CP.

According to a recent study, more parents are aware of how CP affects their child’s cognitive abilities [52]. However, many lack sufficient information about their child’s intellectual capabilities [53]. Most parents recognize the importance of psychiatric treatment and have noticed positive changes in their children after receiving it [54]. Parents must have education and information about their child’s condition and specific needs to notice changes in their mental abilities. Caregivers of children with CP are eager to learn as much as possible about their child’s condition, including their prognosis and the need for rehabilitation intervention [55,56]. Unfortunately, most participants of the current study did not receive any explanation about their child’s mental health, which can negatively impact their occupational engagement, productivity, social participation, and satisfaction. In this study, 64.7% of caregivers noticed that CP affects their child’s mental abilities. A study reported several social and physical risk factors for mental health disorders in children with CP [57].

According to a recent study, many caregivers believe that children with CP struggle with cognitive difficulties during their daily activities [58]. Specifically, 60.3% of participants noted that the physical and mental challenges associated with the condition can negatively impact learning. In 2023, a study conducted by [59] suggested that children with CP may experience weak cognitive abilities and physical limitations, making it challenging for them to focus and to engage in lessons. The study also found that nearly half of the parents surveyed noticed a decrease in their child’s cognitive abilities during group activities, with 60.3% of those parents confirming that their child with CP faces many restrictions during daily activities. According to a study by [60], children with CP commonly encounter obstacles when participating in family activities because of physical limitations such as low muscle strength and fatigue caused by neuromuscular disorders. This finding has also been reinforced by other research studies [61].

There is concern about caregivers’ employment attendance and whether caring for their children affects their work attendance. More than half of the respondents (51.5%) confirmed they find it challenging to attend work consistently due to the need to accompany their children when they are sick or going through complex situations. A study conducted by [62] found that caring for children with disabilities negatively impacts mothers’ employment, working hours, and income. The more severe the child’s situation, the more likely the mother was to earn less, work fewer hours, or resign.

In this study, 55% of caregivers of children with CP reported satisfaction with their work performance. Caregivers of children with disabilities tend to continue working full-time and to engage in paid labor to provide adequate care for their children. A study confirmed that caring for children with disabilities involves indirect economic costs that place a financial burden on families [63]. Almost half of the participants (49.3%) in this study reported that caring for a child with CP causes a financial burden on the family.

Numerous studies from around the world, including some from the Middle East, have shown that caring for a child with a disability limits mothers’ socialization and employment opportunities and reduces their quality of life for various reasons, such as financial strain due to the high cost of medication and specialized care or from experiencing social stigma [36,37,38,64].

### 4.1. Mental Health Care and Social Stigma

Caring for children with CP can present various challenges, including psychological difficulties that can impact the children and their caregivers. These children often experience reduced motor function and struggle with health, sensory, and perceptual issues, along with limitations in self-care activities like feeding, dressing, bathing, and mobility [65]. Additionally, they may be at risk of seizures, cognitive challenges, and behavioral, learning, and emotional impairments [66]. Caregivers may need to seek psychological support for their children, but they may encounter stigma and prejudice regarding this care. The findings of the current study show that 41.0% of the caregivers had experienced stigma and prejudice, especially regarding their psychological care. This stigma can also hinder the overall development of the child with CP and prevent caregivers from participating in social activities and socializing with their other children. Unfortunately, this stigma can even lead to families hiding their children with disabilities, making it difficult for them to find acceptance in society. This proves that stigmatization affects not just those children with CP but their caregivers as well [67,68,69]. There is evidence in the literature that explores the link between social stigma and mental health care for children with CP. A study conducted by [62] found a significant correlation between the psychosocial burden experienced by caregivers and the social stigma they suffered surrounding CP. Another study highlights the feeling of guilt some caregivers experience, along with social stigma, as factors contributing to their decision to delay or avoid seeking mental health services for their children [70,71]. Many studies have shown that disabled children and their families face ongoing discrimination and social stigma [72,73,74]. This can lead to isolation for both mother and child. Similarly to a study in Lebanon [75] that linked social isolation to stigma, research by [76] found that mothers experiencing stigma felt more stressed, which limited their children’s interaction with peers. The need for broader access to facilities and support, not just in major cities, in Saudi Arabia is highlighted by [67,77]. These findings further suggest that family-centered approaches, where parents and professionals work together, are beneficial.

Some caregivers may find that providing care is a taxing, heavy duty that negatively affects their physical and mental health [78,79]. The mental health of family members and caregivers of children with CP has been linked to various mental health problems [80,81]. Numerous studies have examined the factors that influence the psychological well-being of caregivers of children with disabilities and their quality of life [26,81].

CP encompasses various illnesses with varying degrees of disability, impacting the need for psychological care and caregiver burden and affecting children’s emotional and cognitive development. The impact of the types of CP on children’s psychological well-being is enormous. According to [82], spastic CP, for instance, might lead to frustration with movement limitations, while ataxic CP could cause difficulties with coordination and balance, affecting self-esteem. The level of physical impairment can impact a child’s ability to participate in activities and to socialize. This can lead to feelings of isolation, depression, and anxiety [45]. Children with severe CP might experience additional challenges due to communication difficulties. The psychological care needs might involve individual or group therapy to address emotional challenges like anxiety, depression, or low self-esteem. Children also might need help from their therapists to develop coping mechanisms for dealing with frustrations and limitations caused by CP. They also need training to enhance their social skills, particularly for children who struggle with communication or social interactions caused by the disorder. The impact of the types of CP on caregivers could be due to the care parents provide to a child with CP, which can be emotionally draining, leading to stress, anxiety, and even depression [83]. This is in addition to the physical burden caused by the daily care routines, especially for children with severe CP. This can lead to fatigue and physical health concerns. To tackle these burdens there is an increased need for support groups that can be invaluable for caregivers, providing a platform to share experiences, find emotional support, and learn helpful strategies [84]. Parents/caregivers need access to respite care services that can offer breaks for caregivers, allowing them to recharge and to avoid burnout. Financial assistance programs can help alleviate financial pressure. Following this comprehensive approach will significantly improve both the child’s quality of life and the caregiver’s ability to provide effective long-term care.

### 4.2. Limitations of the Study

The study primarily focused on caregiver perceptions in Saudi Arabia. A direct assessment of the children’s mental health needs is required. Further research could explore the effectiveness of interventions aimed at improving caregiver awareness of mental health services and reducing social stigma. While self-reported questionnaires offer valuable insights from parents/caregivers of children with CP, they can also be susceptible to social desirability bias. This means that participants might answer in the way they believe is expected or “correct”, rather than in a way that reflects their actual experiences, particularly if they are affected by social stigma concerning their children’s condition. Future research could incorporate a more comprehensive picture by using additional methods, such as observing how children interact with their environment or by using objective assessments.

## 5. Conclusions

This study highlights the critical need for a two-pronged approach to supporting children with CP and their caregivers. While children with CP benefit from psychological interventions that address their emotional well-being and social interaction, caregivers require vigorous support systems to manage stress, improve their coping mechanisms, and enhance their overall well-being. The findings of this study suggest that there is a lack of knowledge concerning the importance of psychological care for their children, while a number of participants expressed concerns about social disapproval concerning the psychological services that they seek for their children with CP. The findings also highlighted that the disorder caused the family financial strain and restricted their participation in social life, including socializing and pursuing hobbies. This study highlights the importance of psychological care for children with CP and its positive impact on their occupational performance. These results emphasize the importance of developing comprehensive care programs that integrate child-focused psychological interventions with caregiver support initiatives. Intervention programs are needed to foster a holistic approach to improving the quality of life for both children with CP and their caregivers. Social stigma surrounding mental health, which has been explored in this study, caused a huge barrier for parents/caregivers in seeking help for their children with CP. Researchers need to explore ways to dismantle these negative perceptions within communities. Awareness campaigns to praise mental well-being or educational programs that foster empathy and understanding will be beneficial. By tackling stigma head-on, researchers can create a more supportive environment where children can get the care they need without fear of judgment. Future research should explore the long-term effects of these interventions and investigate the most effective methods for delivering support services in diverse communities.

## Figures and Tables

**Table 1:** Demographic characteristics of the participants.

Characteristics			Frequency	Percent
Caregivers	Primary caregiver	Mother	192	64.0
		Father	65	21.7
		External assistant (servant)	43	14.3
	The educational level of the primary caregiver	Illiterate	55	18.3
		Primary education	119	39.7
		Higher education (bachelor’s and above)	126	42.0
	Employment status	Employed	136	45.3
		Non-employed	164	54.7
Children	Age	<6 years	117	39.0
		6–10 years	131	43.7
		>10 years	52	17.3
	Gender	Boys	150	50
		Girls	150	50

**Table 2:** The participants’ response to the survey.

Item	Responses	Frequency	Percent
Do you know the meaning of psychiatric care?	Yes	174	58.0
	No	126	42.0
Access to psychiatric care			
Has the child had any psychiatric care before?	Yes	87	29.0
	No	213	71.0
Did the child have any referral to a psychologist?	Yes	121	40.3
	No	179	59.7
Do you think that psychiatric care is mainly linked to social stigma?	Yes	123	41.0
	No	177	59.0
A child’s cognitive ability			
Has a full explanation of your child’s intellectual disability been discussed with you before?	Yes	140	46.7
	No	157	52.3
	Other	3	1.0
Do you think that cerebral palsy can affect your child’s cognition?	Yes	194	64.7
	No	104	34.7
	Other	2	0.7
Do you notice a reduction in your child’s cognitive abilities when engaging with others?	Yes	145	48.3
	No	152	50.7
	Other	3	1.0
Do you think that your child faces restrictions during his performance of activities?	Yes	181	60.3
	No	116	38.7
	Other	3	1.0
Do you think your child’s occupational performance is mainly affected due to a reduction in their cognitive level?	Yes	166	55.3
	No	129	43.0
	Other	5	1.7
Perceived benefit of psychiatric care			
If your child had access to psychiatric care, did you notice any improvement?	Yes	61	70.1
	No	26	29.9
Do you think your child’s performance can increase following psychiatric care?	Yes	197	65.7
	No	102	34.0
	Other	1	0.3
Do you need to deal with a psychologist to facilitate your understanding of your child’s behavior?	Yes	151	50.3
	No	149	49.7
The impact of a child’s condition on the caregiver’s life			
Are you employed?	Yes	136	45.3
	No	164	54.7
If the previous question is answered by yes, does taking care of your child impact your attendance at work?	Yes	70	51.5
	No	66	48.5
Are you satisfied with your occupational role and domestic role?	Yes	165	55.0
	No	135	45.0
Does your child’s condition cause a financial burden for the family?	Yes	148	49.3
	No	152	50.7
Does your child’s condition affect your social life (visiting interest)?	Yes	147	49.0
	No	153	51.0

## Data Availability

Data supporting the findings of this study are available from the corresponding author upon request.
